# Silicon Hybridization for the Preparation of Room-Temperature Curing and High-Temperature-Resistant Epoxy Resin

**DOI:** 10.3390/polym16050634

**Published:** 2024-02-27

**Authors:** Liping Rong, Jiaqi Su, Zhiguo Li, Xiaohui Liu, Dayong Zhang, Jinhua Zhu, Xin Li, Ying Zhao, Changhong Mi, Xianzhi Kong, Gang Wang

**Affiliations:** 1 Institute of Petrochemistry, Heilongjiang Academy of Sciences, Harbin 150040, China; rlpshy002@163.com (L.R.); liuxiaohui0201@aliyun.com (X.L.); 12zdy@163.com (D.Z.); zhujinhua81@126.com (J.Z.); lixin8503@163.com (X.L.); zhaoying62@sina.com (Y.Z.); changhongmi@163.com (C.M.); 2 Key Laboratory of Bio-based Materials Science & Technology, Ministry of Education, College of Material Science and Engineering, Northeast Forestry University, Harbin 150040, China; sujiaqi@nefu.edu.cn (J.S.); lizgmse@nefu.edu.cn (Z.L.)

**Keywords:** silicon-hybridized epoxy resin, room-temperature curing, heat resistance, sol–gel method, adhesive

## Abstract

Specialized epoxy resin, capable of achieving room-temperature profound curing and sustaining prolonged exposure to high-temperature environments, stands as a pivotal material in modern high-end manufacturing sectors including aerospace, marine equipment fabrication, machinery production, and the electronics industry. Herein, a silicon-hybridized epoxy resin, amenable to room-temperature curing and designed for high-temperature applications, was synthesized using a sol–gel methodology with silicate esters and silane coupling agents serving as silicon sources. Resin characterization indicates a uniform distribution of silicon elements in the obtained silicon hybrid epoxy resin. In comparison to the non-hybridized epoxy resin, notable improvements are observed in room-temperature curing performance, heat resistance, and mechanical strength.

## 1. Introduction

Epoxy resin represents a class of thermosetting polymer materials containing a minimum of two active epoxy groups per molecule. Renowned for its exceptional mechanical, adhesive, electrical insulation, and chemical stability properties, epoxy resin is widely employed in diverse applications, such as adhesives, coatings, insulating materials, and advanced composite materials [1,2,3,4]. As the utilization of epoxy resin-based composite materials proliferates in the realm of high-end manufacturing, heightened expectations are directed towards their thermal endurance performance [5]. Nevertheless, epoxy resins typically necessitate heat-induced curing to elevate the degree of cure, thereby augmenting crosslink density and enhancing heat resistance. However, challenges arise in certain domains such as aerospace, marine equipment manufacturing, machinery production, and the electronics industry, where the application of heat curing is impractical for certain large components, parts intolerant to high temperatures, and heat-sensitive components [6,7]. For instance, within the domain of epoxy resin-based heat-resistant composite materials, structural assembly requires room-temperature curing alongside the incorporation of liquid shims, compensation fillers, aircraft structural adhesives, precision parts adhesives, electronic potting adhesives, and other applications, all of which mandate the utilization of high-temperature-resistant epoxy resin materials at specified levels. Hence, the development of a novel system that undergoes room-temperature curing and exhibits high-temperature functionality represents a crucial research direction for the high-performance advancement of epoxy resins, and has emerged as a focal point in current research endeavors within this field.

Currently, a research methodology to enhance the room-temperature curing proficiency and high-temperature durability of epoxy resins involves the investigation of curing agents. Specifically, this entails the utilization of ambient-temperature curing agents containing thermally resistant functional groups for the curing process of epoxy resins. Yuan et al. [8] employed 4,4′-diaminodiphenylmethane (DDM), 2,2-Bis[4-(4-aminophenoxy) phenyl] propane (BAPP), or 4,4′-(hexafluoroisopropylidene) bis(p-phenoxy) diphenylamine (HFBAPP) as curing agents to cure bisphenol A-type epoxy resin (E-51) under the catalysis of acrylic acid (HAA). Benefiting from the introduction of a significant number of benzene ring structures by the curing agents into the crosslinked network, the resulting products demonstrated elevated thermal stability, higher glass-transition temperatures, and excellent high tensile strength. Xu et al. [9] fabricated a novel self-exothermic curing agent via the combination of DDM and acrylic acid, which demonstrates the capability to efficiently cure epoxy resin at room temperature within a time of 3 h. Moreover, the integration of composite short hemp fibers into room-temperature-cured epoxy resin yields rapidly forming high-performance composites. Zheng et al. [10] prepared a curing agent for room-temperature-curable E51 by mixing 1-hexyl-3-methylimidazolium tetrachloroferrate ([C_6_mim]FeCl_4_) with various amine blends at different mass ratios. The E51 cured with this curing agent exhibited excellent thermal stability, surpassing the reference E51 epoxy resin by at least 30 °C.

As the main component of epoxy resins, theoretically, modifying the resin structure can lead to improved thermal stability and enhanced room-temperature curability. However, due to limitations in the synthesis processes and cost considerations, reports in this regard are relatively scarce. Zhang et al. [11] attempted the synthesis of a three-functional epoxy resin with improved performance using resorcinol diglycidyl ether and 1,1,1-tris(4-hydroxyphenyl) ethane as raw materials. Reactivity studies with various curing agents revealed enhanced curing degree, glass-transition temperature, and thermal-resistance properties in the resulting products based on this resin. The observed improvements can be attributed to the incorporation of a heat-resistant functional group (phenyl ring) into the epoxy resin structure, thereby increasing both the heat resistance functional groups and the functionality of the epoxy resin. This dual enhancement contributes to an elevated cross-linking density of the cured material, consequently improving the thermal resistance of the room-temperature-cured epoxy resin. Generally, inorganic siloxanes exhibit high thermal stability. Introducing siloxanes into epoxy resins, as opposed to aromatic rings, is considered a more promising method to significantly enhance the high-temperature resistance of epoxy resins. In this study, a siloxane-hybridized epoxy resin was developed by incorporating the higher-bond-energy Si-O-Si bonds into the molecular structure of epoxy resin and introducing additional epoxy groups through a silane coupling agent. Ethyl orthosilicate (TEOS) and silane coupling agent (KH560) were selected as silicon sources. In the molecular structure of TEOS, the silicon atom is coordinated with four ethyl groups, forming a tetrahedral structure. Due to the relatively low electronegativity of the silicon atom, the bond between silicon and oxygen atom is characterized by weak polarity, rendering it susceptible to hydrolysis, yielding Si-OH moieties that subsequently undergo condensation to form Si-O-Si gel. Consequently, the sol–gel reaction conditions utilizing TEOS as the precursor exhibit mildness and are readily controllable. The choice of KH560 as the silane coupling agent is predicated upon its chemical structure, which encompasses Si-O-CH_3_ and epoxy functionalities. KH560, in conjunction with TEOS, facilitates the sol–gel reaction, yielding silica sol. The incorporation of epoxy groups into the silica sol structure serves to further augment the compatibility between silica sol and epoxy resin. The resin was prepared, and its performance was evaluated with three different curing agents: polyamide 300#, polyetheramine D230, and triethylenetetramine (TETA). The impact of siloxane hybridization on the room-temperature curing ability and high-temperature resistance of the epoxy resin was systematically investigated.

In this study, rather than employing conventional methods of incorporating SiO_2_ particles to modify epoxy resins, a novel approach involving the integration of the Si-O-Si-inorganic network into epoxy resins was pursued. When commercial SiO_2_ nanoparticles are simply blended with epoxy resins, achieving a homogeneous dispersion of the nanoparticles poses a considerable challenge, often resulting in agglomeration issues. Such agglomeration phenomena invariably lead to a deterioration in the performance characteristics of epoxy resins. The modification of epoxy resin through siloxane hybridization introduces Si-OH, Si-O-Si, and Si-O-C bonds into the epoxy resin matrix. These bonds play a pivotal role in facilitating room-temperature curing of the epoxy resin and enhancing the high-temperature resistance of the cured material. Notably, high-temperature-resistant adhesives utilized in aerospace, machinery, and electronics applications frequently necessitate ambient curing conditions due to factors such as the large dimensions of assembly parts, the temperature sensitivity of precision components, and notable differences in the coefficients of thermal expansion among the bonding materials. Therefore, this modification of epoxy resin through siloxane hybridization notably improved its room-temperature curing performance. This method provides a novel approach for the research and development of room-temperature-cured, high-temperature-resistant epoxy resins, demonstrating broad prospects for practical applications.

## 2. Experiment

### 2.1. Materials

The epoxy resin (E-51, CP) was used as received from Nantong Star Synthetic Material Co., Ltd. (Nantong, China); Tetraethoxysilane (TEOS) (AR), isopropanol (AR) and triethylne tetramine (TETA) (AR) were obtained from Tianjin Komio Chemical Reagent Co., Ltd. (Tianjin, China). Polyamide (300#, CP) was obtained from Tianjin Haiyan Chemical Co., Ltd. (Tianjin, China). Dibutyltin dilaurate as catalyst and polyether amine (D230, AR) were purchased from Shanghai Macklin Biochemical Technology Co., Ltd. (Shanghai, China). Silane coupling agent (KH-560, CP) was purchased Nanjing Shuguang Coupling Agent Co., Ltd. (Nanjing, China). Hydrochloric acid (37%, AR) was obtained from Xilong Science Co., Ltd. (Shantou, China). Distilled water (CP) was used as received from the Harbin Thermal Distilled Water Plant (Harbin, China).

### 2.2. Methods

#### 2.2.1. Preparation of Silica Sol

A total of 0.61 g Isopropanol, 2.08 g TEOS and 7.08 g KH-560 were added sequentially in a three-necked flask and stirred for 30 min at 30 °C. Then, 0.06 g dibutyltin dilaurate and 2.2 g hydrochloric acid aqueous solution (0.45 mol/L) were added, and the system was heated to 60 °C for 4 h to react and obtain a SiO_2_ sols. The role of the catalyst is to promote hydrolysis in TEOS.

#### 2.2.2. Preparation of SiO_2_ Hybrid Epoxy Resin

A total of 200 g of epoxy resin (EP) was added to SiO_2_ sols synthesized as above, under vacuum (−0.098 MPa); this then underwent rotary evaporation at 75 °C for 15 min, rotary evaporation at 90 °C for 30 min, and was then cooled to room temperature; a SiO_2_ hybrid epoxy resin was obtained.

#### 2.2.3. Process of Curing

According to the calculation of the equivalent ratio of the epoxy group to the amine group, the above-synthesized silicone-hybridized epoxy resin (Section 2.2.2) and unhybridized epoxy resin were mixed with polyamide 300#, polyether amine D230, and TETA to prepare the specimens, respectively. This mixture was then cured at room temperature for 7 days and tested.

There are no specific requirements for size and shape of IR, SEM, EDS, DSC, and TG test specimens. The size and shape of the shear strength, peel strength, and DMA test specimens need to be prepared as shown in Section 2.3 in this document.

### 2.3. Tests and Characterization

The surface-functional groups of epoxy resins and hybridized epoxy resins were determined using an IRTracer-100 Fourier infrared spectrometer from Shimadzu, Kyoto, Japan, with a scanning range from 450 cm^−1^ to 4000 cm^−1^. The test specimen was applied to the surface of the KBr thin slices, and a resolution and number of scans of 4 cm^−1^ and 32 times/min, respectively. The cured resin samples were brittle and fractured under liquid nitrogen, and a gold film was sprayed on the freshly fractured surface. A JSM IT300 scanning electron microscope from Nippon Electron Co. (Tokyo, Japan) was used to observe the micro-morphology of the fracture surface. At the same time, the Si element in the test specimen was labeled using an Energy-Dispersive Spectrometer (EDS).

DSC was used to test the curing reactivity of different curing systems. The specimen mass was 5 mg–10 mg, the temperature range was from 25 °C to 250 °C, and the heating rate was 10 °C/min. Initial curing temperatures of epoxy and silicon-hybridized epoxy resins were derived from DSC curves. The above specimens were cured at room temperature for 7 days and then tested by DSC under the same conditions. The chemical reaction heat before and after resin curing were calculated by DSC curve. According to “HB7614-1998 [12] Differential Scanning Calorimetry (DSC) Test Method for Curing Degree of Composite Resin Matrix”, the curing degree was calculated as follows:

$$\alpha =\frac{H_{T}-H_{R}}{H_{T}}\times 100$$

Here, *α* stands for the curing degrees (%), *H_T_* for the total chemical reaction heat (mJ/mg), and *H_R_* for the residual chemical reaction heat (mJ/mg). Mechanical property tests were conducted using the INSTRON-5696 mechanical testing machine from the U.S. company Yingstron (Norwood, MA, the USA). Chromic-acid-treated aluminum alloy specimens measuring 3 mm × 20 mm × 60 mm were bonded with epoxy resins of different curing systems, creating a bonding area of 15 mm × 20 mm. The bonded specimens were cured at room temperature for 7 days with a pressure of 0.04 MPa, and then tested for shear strength at room temperature, 80 °C, and 120 °C following “HB5164-1981 [13] Test Method for Tensile Shear Strength of Adhesively Bonded Metals”. The test speed was 10 mm/min, and the tensile shear strength was calculated by dividing the maximum breaking load force by the bond area. Aluminum alloy specimens anodized by the “HB/Z197-1991 [14] Structural Bonding Aluminum Alloy Phosphoric Acid Anodizing Process Specification” method were bonded through epoxy resins of different curing systems. The size of the specimens was 3 mm × 25 mm × 200 mm and 0.3 mm × 25 mm × 200 mm, respectively. After curing the bonded specimens for 7 days at room temperature with a pressure of 0.04 MPa, the peel strength of the bonded specimens was tested following “GJB446-1988 [15] Test Method for 90° Peel Strength of Adhesives (Metal to Metal)”. The test speed was 100 mm/min, and the peel strength was calculated by dividing the average breaking load force by the width of specimen.

The epoxy resins with different curing agent types before and after hybridization were cured at room temperature for 7 days and made into 3 mm × 10 mm × 50 mm test specimens, which were tested using Q400 dynamic mechanical analyzer from TA, New Castle, DE, USA. The three-point bending test mode was used, the vibration frequency was 1 Hz, the temperature range was from room temperature to 150 °C, and the heating rate was 10 °C/min. A temperature dependence curve of the test sample’s loss factor (tanδ) was obtained. The temperature corresponding to the maximum value of tanδ was the specimen’s glass-transition temperature (Tg). Cured and hybrid epoxy samples were tested using a thermogravimetric analysis (TGA55) from TA, New Castle, DE, USA. The specimen mass was 5 mg–10 mg, the temperature ramp rate was set at 10 °C/min in an air atmosphere, and the temperature range was from room temperature to 750 °C. Mass versus temperature curves were obtained.

## 3. Results and Discussion

### 3.1. Structural Analysis

We assessed the extent of curing, shear strength, and weight loss of silicon-hybridized epoxy resins and non-hybridized epoxy resins under varying conditions including room temperature, 80 °C, and 120 °C. Additionally, weight loss at 400 °C was measured as a primary comparative parameter. These analyses were conducted utilizing DSC, the electronic tensile machine, and TG, respectively. The findings are presented in Table 1.

**Figure 1:**
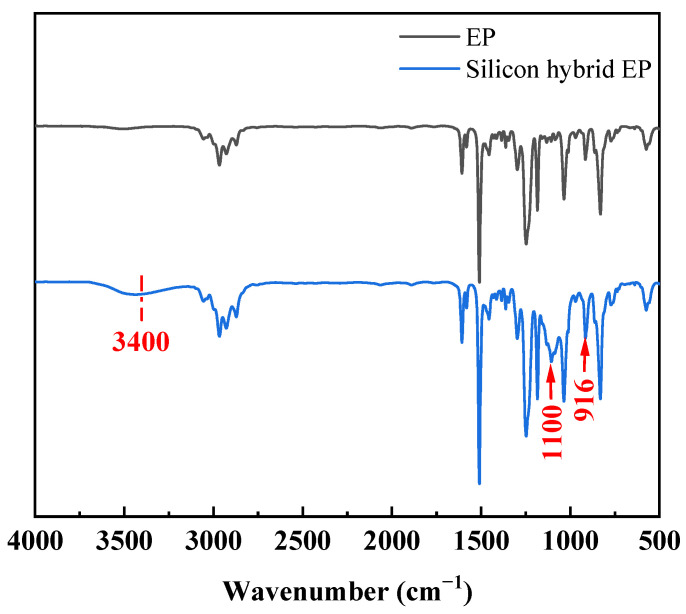
FTIR spectra of EP and silicon hybrid EP.

The infrared spectra of epoxy resin (EP) before and after silicon hybridization are shown in Figure 1. The IR spectra of the silicon-hybridized epoxy resin showed characteristic absorption peaks at 1100 cm^−1^ for Si-O-Si and Si-O-C bonds, indicating that Si-OH produced by TEOS hydrolysis was further condensed to Si-O-Si bonds, as well as reacted with -OH in the epoxy resin to introduce SiO_2_ inorganic network into the molecular structure of the epoxy resin. At 3400 cm^−1^ was the characteristic absorption peak of Si-OH, indicating that not all Si-OH condensation reactions occurred [16,17]. During the reaction process, the epoxy group absorption peak 916 cm^−1^ did not change, indicating that the process of preparing silicon-hybridized epoxy resin has little effect on the epoxy group [18].

**Figure 2:**
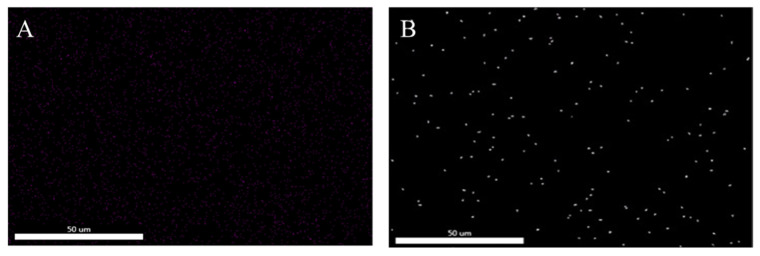
The EDS energy spectrum of (**A**) EP and (**B**) silicon hybrid EP.

**Figure 3:**
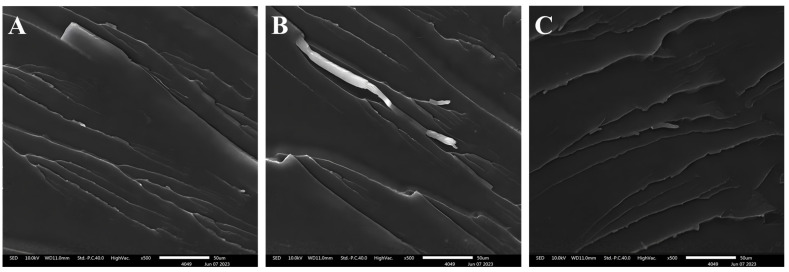
SEM images of EP cured with different curing agents: (**A**) 300#, (**B**) D230, (**C**) TETA.

Figure 2 shows the EDS energy spectrum analysis of the epoxy resin before and after silicon hybridization. The white dots in Figure 2B were the Si element distribution, indicating that the Si element was introduced into the structure of the hybridized epoxy resin and was more uniformly dispersed. SEM photographs of the silicon-hybridized epoxy resins cured at room temperature with three different curing agents are shown in Figure 3, which showed ductile fracture; moreover, no agglomeration or precipitation of SiO_2_ particles was found in any of the three SEM photographs, indicating that the SiO_2_ was uniformly dispersed in the hybridized resins. SiO_2_ particles cannot be seen in SEM or TEM. This is due to the fact that in silicon-hybridized epoxy resins, SiO_2_ exists in the epoxy resin structure as an inorganic network of Si-O-Si-.

**Figure 4:**
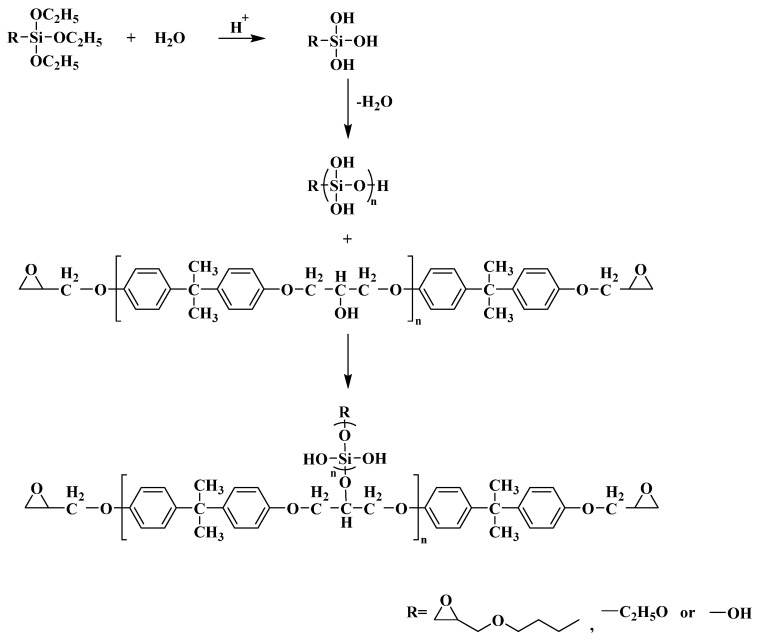
Scheme of synthesis reaction mechanism of silicon hybrid resin.

Combining the structural characterization results of IR, EDS, and SEM of the above reaction process, it can be seen that the structural formula of the synthesis reaction of the silicon hybrid resin was roughly shown in Figure 4.

### 3.2. Curing Reactivity Analysis

**Figure 5:**
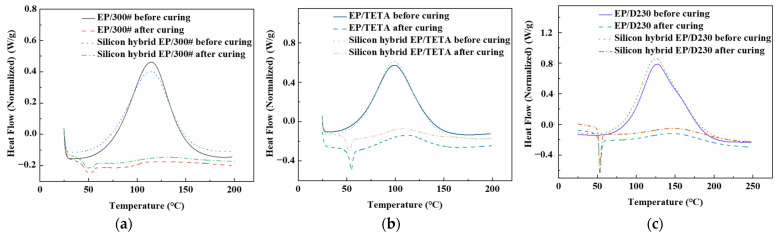
DSC curves of different component epoxy resins before and after curing: (**a**) EP/300# and silicon hybrid EP/300#, (**b**) EP/D230 and silicon hybrid EP/D230, (**c**) EP/TETA and silicon hybrid EP/TETA.

The curing reactivity of the resin is closely related to its performance. The higher the curing reactivity, the higher the degree of room-temperature curing, and the better its performance is expressed. Following the test standard “HB7614-1998 Differential Scanning Calorimetry (DSC) Test Method for Curing Degree of Composite Resin Matrix”, DSC tests were conducted on the specimens before and after curing of three different curing agent/resin systems, and the experimental results are shown in Figure 5. The degree of cure of the different curing agent/resin systems and the starting cure temperatures shown in the DSC curves are shown in Table 2.

Table 2 showed that when using the same curing agent, the curing degree of silicon-hybridized epoxy resin was increased compared to unhybridized epoxy resin, and the onset curing temperature was reduced. This was due to the presence of uncondensed Si-OH in the silicon-hybridized epoxy resin, and the -OH could promote the ring-opening reaction of the epoxy group [19,20]. As a result, the degree of curing and curing reactivity of hybridized epoxy resins under room-temperature conditions can be improved, laying the foundation for improving performance after room-temperature curing.

### 3.3. Bonding Performance Analysis

The adhesive properties of room-temperature-cured silicon hybrid epoxy resins were investigated by testing the tensile shear strength at different temperature conditions and peel strength at room temperature.

#### 3.3.1. Tensile Shear Strength

**Figure 6:**
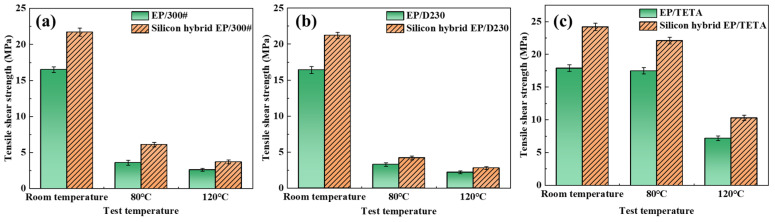
Tensile shear strength of room-temperature-cured EP and silicon hybridization EP with different curing agent compositions: (**a**) 300#, (**b**) D230, (**c**) TETA.

Aluminum sheets were bonded with EP/silicon-hybridized EP with different curing agent compositions, and the tensile shear strength of the bonded specimens was tested at room temperature, 80 °C, and 120 °C. The experimental results are shown in Figure 6.

The experimental results showed that the tensile shear strengths of the three different room-temperature curing agents curing silicon-hybridized epoxy resins were higher than those of the corresponding unhybridized epoxy resins at room temperature, 80 °C, and 120 °C. This is because in the structure of silicon-hybridized epoxy resin, the -Si-O-Si-bonds were uniformly dispersed in the form of an inorganic network in the organic structure of the epoxy resin. When exposed to external forces, SiO_2_ can absorb and transfer part of the internal stresses so that the inorganic network structure in the hybridized system plays a toughening role in enhancing the room-temperature shear strength of the silicon-hybridized epoxy resin. Meanwhile, the higher bond energies of the Si-O-Si and Si-O-C bonds resulted in better heat resistance of the structure. The toughening effect synergizes with heat resistance, improving the high-temperature shear strength.

#### 3.3.2. Peel Strength

**Figure 7:**
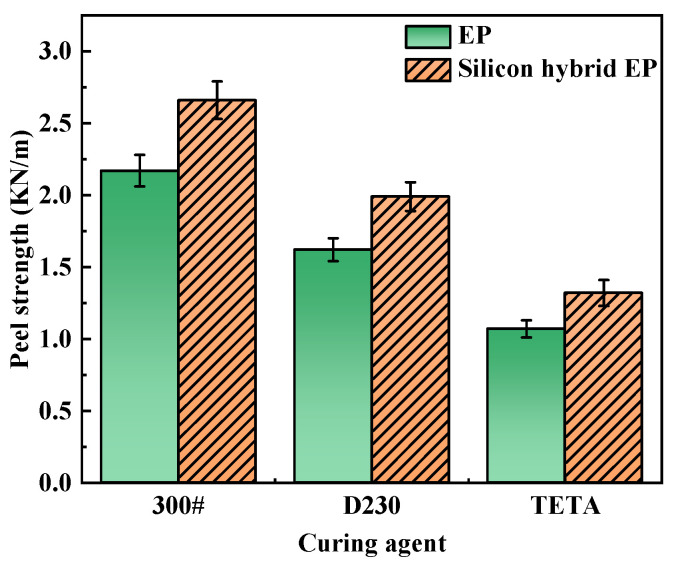
Peel strength of EP and silicon hybrid EP cured by different curing agents.

The 90° peel strength of the bonded specimens was tested using EP and silicon-hybridized EP bonded specimens cured at room temperature with three different curing agents. The experimental results were shown in Figure 7.

The experimental results showed that the peel strengths of the three different curing agents for room-temperature-cured silicon-hybridized EP were higher than those of the corresponding unhybridized EP. In the exact mechanism of the tensile shear strength, the SiO_2_ inorganic network structure in the hybridized system acted as a toughening agent to enhance the peel strength of the silicon-hybridized epoxy resin.

### 3.4. Heat Resistance Analysis

The study of the high-temperature resistance properties of room-temperature-cured silicon-hybridized epoxy resins is of great significance as a guide for applying of room-temperature-cured high-temperature-resistant materials. In this study, three different curing agents were used to cure epoxy resins before and after silicon hybridization at room temperature, and the glass-transition temperature and heat loss properties of the cured specimens were analyzed.

#### 3.4.1. Glass-Transition Temperature

**Figure 8:**
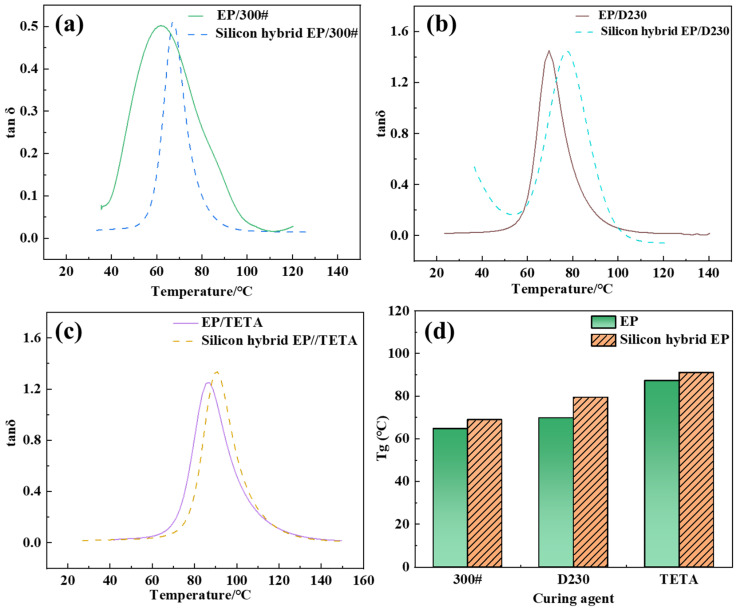
DMA curves of EP and silicon hybrid EP cured by different curing agents: (**a**) 300#, (**b**) D230, (**c**) TETA, (**d**) Tg of different curing resin systems.

Three curing agents obtained the DMA curve to cure the epoxy resin before and after room-temperature silicon hybridization. The temperature corresponding to the maximum value of the loss factor tanδ in the curve was the test specimen’s glass-transition temperature (Tg); the experimental results are shown in Figure 8.

The DMA curves showed that the Tg of the three different curing agents for room-temperature-cured silicon-hybridized epoxy resins was higher than that of the corresponding unhybridized epoxy resins. This may be due to the complex network structure of Si-O-Si and the large spatial site resistance limiting the movement of the epoxy chain segments [21,22]. On the other hand, due to the further reaction between the residual Si-OH in the Si-O-Si inorganic network and the -OH and epoxy groups in the epoxy resin during the heating test process (or during high-temperature use), the inorganic network was more closely bonded to the epoxy resin, and the organic–inorganic hybridization in the structure of the cured object was more complete. Combining these two factors led to a higher glass-transition temperature for silicon hybrid epoxy resins than unhybridized ones.

#### 3.4.2. Thermal Weight Loss

**Figure 9:**
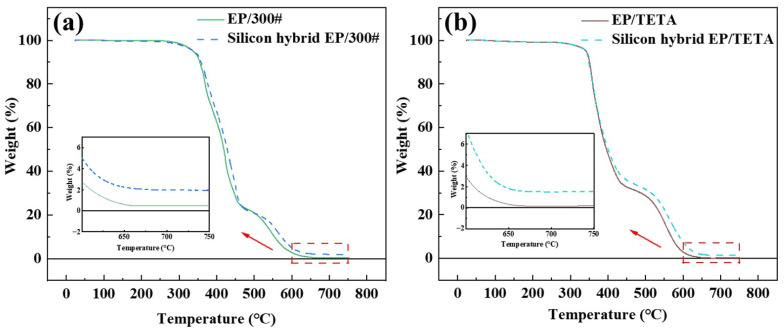

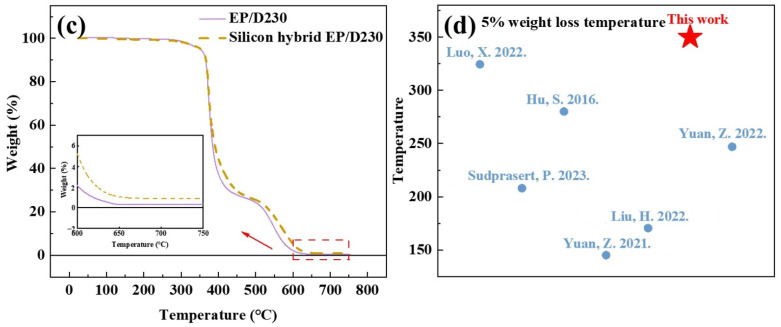
TG curves of EP and silicon hybrid EP cured by different curing agents, (**a**) 300#, (**b**) D230, and (**c**) TETA. (**d**) Comparison of 5% weight loss temperature of room-temperature-cured epoxy resins, Hu, S. 2016 [6], Yuan, Z. 2021 [8], Lou, X. 2022 [23], Liu, H. 2022 [24], Sudprasert, P. 2023 [25], Yuan, Z. 2022 [26].

The TG curves of epoxy resins before and after silicon hybridization cured at room temperature with three different curing agents were tested, and the results are shown in Figure 9. The temperature at which each specimen lost 5% weight and the weight loss rate at 400 °C are shown in Table 3.

Table 3 shows room-temperature-cured silicon-hybridized epoxy resin compared for hybridization; the temperature of 5% weight loss was found as a result of a slight increase in temperature, whereas 400 °C weight loss was found as the temperature decreased further. Figure 9 indicates that the weight loss of silicon-hybridized epoxy resin was lower than that of unhybridized epoxy resin after 400 °C, demonstrating that the enhancement of thermal stability of silicon-hybridized epoxy resin at high temperatures was relatively obvious. Compared to other room-temperature-cured epoxy resins, the 5% weight loss temperature of this work was higher, implying a high initial decomposition temperature; this indicates that the heat resistance of the epoxy resin in this work was significant.

The TG stability of a resin is mainly determined by its chemical structure. The introduction of Si-O-Si and Si-O-C bonds with higher bond energies brought about the presence of a nano-SiO_2_ inorganic structure in the hybridized epoxy resin. The SiO_2_ inorganic structure played a protective role in the cross-linking structure during the heat loss test, which resulted in a significant reduction in the high-temperature weight loss of the silicon-hybridized epoxy resin, and an improvement in the high-temperature thermal stability performance [27]. Meanwhile, with the increase in TG test temperature, Si-OH further condenses and hybridizes the epoxy resin twice, which was another reason the weight loss of the hybridized resin was significantly lower than that of the unhybridized resin in the high-temperature region.

## 4. Conclusions

In order to improve the heat resistance of room-temperature-cured epoxy resin, a silica hybrid epoxy resin was successfully synthesized in this thesis. The results showed that silica was uniformly dispersed in the epoxy resin structure. The curing degree at room temperature, room-temperature shear strength, shear strength at 80 °C, shear strength at 120 °C, and weight loss rate at 400 °C of silicon hybrid EP/300#, silicon hybrid EP/D230, and silicon hybrid EP/TETA demonstrated superior performance compared to EP/300#, EP/D230, and EP/TETA, respectively. Specifically, when utilizing the curing agent 300#, the curing degree at room temperature increased from 80.4% to 85.2%, the room-temperature shear strength elevated from 16.5 MPa to 21.7 MPa, the shear strength at 80 °C rose from 3.6 MPa to 6.1 MPa, and the shear strength at 120 °C improved from 2.6 MPa to 3.7 MPa. Additionally, the weight loss rate at 400 °C decreased from 37.38% to 33.75%.

Compared to the non-hybridized epoxy resin, the hybrid epoxy resin exhibited notable enhancements in room-temperature curing degree, room temperature/high temperature bonding characteristics, and thermal stability properties. The incorporation of silicon into the epoxy resin matrix led to significant improvements in both room-temperature curing and resistance to elevated temperatures. Consequently, silicon-hybridized epoxy resin demonstrates exceptional suitability for various applications, including large-scale assembly parts, precision components vulnerable to high temperatures, and bonding materials where heating is not feasible due to significant disparities in coefficients of linear expansion. The silicon-hybridized epoxy resin in this study opens up new avenues for studying room-temperature-cured high-temperature-resistant epoxy resins.

## Figures and Tables

**Table 1:** Comparison table of key parameters for silicon-hybridized epoxy resins and non-hybridized epoxy resins.

Key Parameters	EP/300#	Silicon Hybrid EP/300#	EP/D230	Silicon Hybrid EP/D230	EP/TETA	Silicon Hybrid EP/TETA
Curing degree at room temperature	80.4	85.2	81.9	86.7	51.6	67.7
Shear strength at room temperature	16.5	21.7	16.4	21.2	17.9	24.2
Shear strength at 80 °C	3.6	6.1	3.3	4.2	17.5	22.1
Shear strength at 120 °C	2.6	3.7	2.2	2.8	7.2	10.3
Weight loss rate at 400 °C	37.38	33.75	62.47	55.53	52.11	47.21

**Table 2:** Curing degree and initial curing temperature of different resin/curing agent systems.

Resin/Curing Agent	EP/300#	Silicon Hybrid EP/300#	EP/D230	Silicon Hybrid EP/D230	EP/TETA	Silicon Hybrid EP/TETA
Curing degree/%	80.4	85.2	81.9	86.7	51.6	67.7
Initial curing temperature/°C	65.3	61.9	73.4	68.3	58.6	54.1

**Table 3:** TG data of different curing resin systems.

Resin/Curing Agent	5% Weight Loss Temperature (°C)	Weight Loss Rate at 400 °C (%)
EP/300#	337.88	37.38
Silicon hybrid EP/300#	338.48	33.75
EP/D230	351.12	62.47
Silicon hybrid EP/D230	352.87	55.53
EP/TETA	341.98	52.11
Silicon hybrid EP/TETA	343.01	47.21

## Data Availability

Data presented in this study are available on request from the corresponding authors.
